# Supplementary material for: CRISPR/Cas13-Mediated Inhibition of EBNA1 for Suppression of Epstein–Barr Virus Transcripts and DNA Load in Nasopharyngeal Carcinoma Cells
Source: Viruses. 2025 Jun 26;17(7):899. doi: 10.3390/v17070899 (PMC12299874; doi:10.3390/v17070899)
Supplement: Supplementary file 1 [file viruses-17-00899-s001.zip › viruses-3719129-supplementary.pdf]

Supplementary Figures

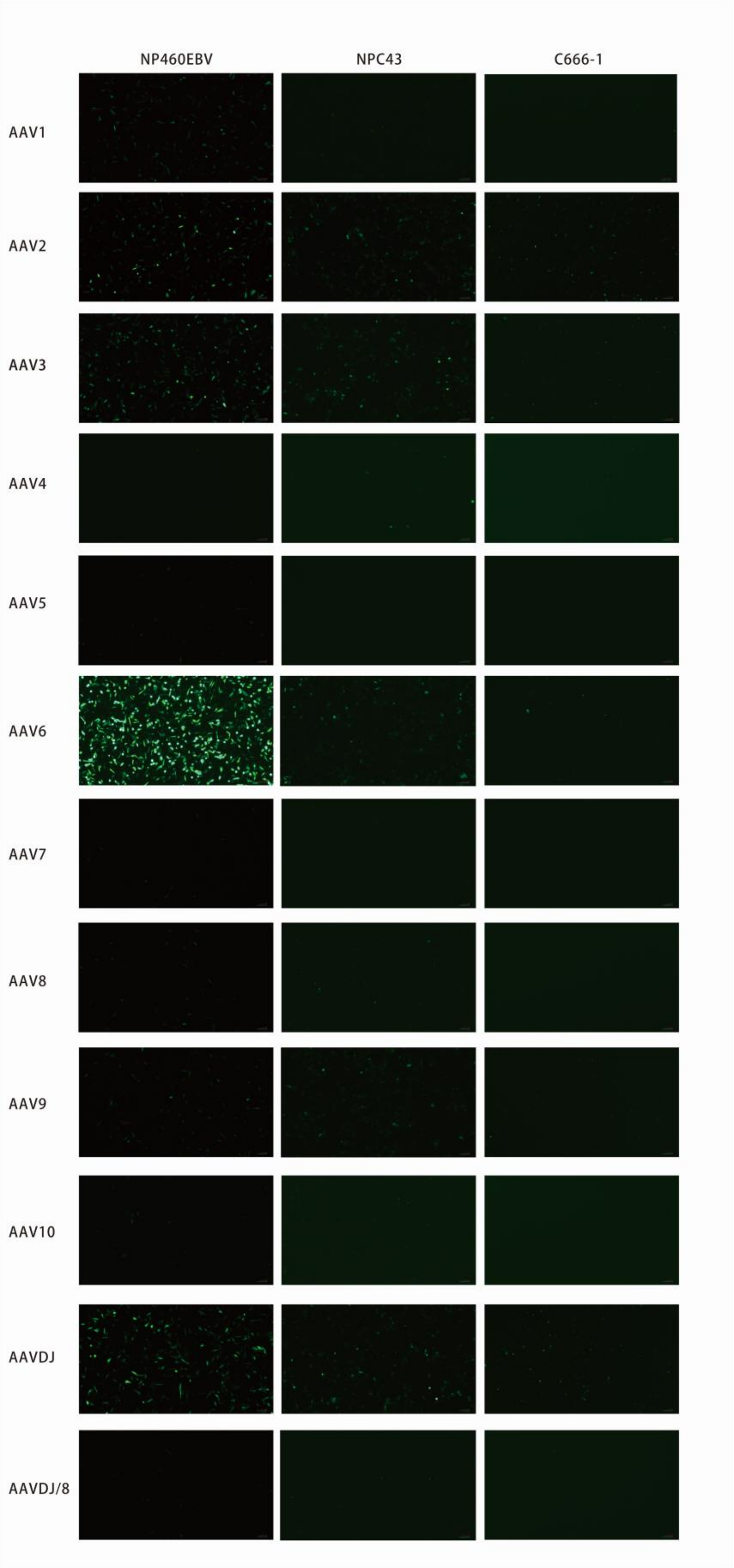

Figure S1. Fluorescent microscopic analysis of AAV serotypes in NPE and NPC cells. The transduction efficiency of various AAV serotypes in the NPE cell line NP460 and the NPC cell lines C666-1 and NPC43 was evaluated using twelve pre-made GFP-expressing AAV serotypes (1, 2, 3, 4, 5, 6, 7, 8, 9, 10, DJ, and DJ/8) at an MOI of 1:10000. The GFP signal was recorded 48 hours after transduction.
